# Water‐Soluble, Enzyme‐Degradable, and Hydrolyzable STAR Particles for Enhanced Drug Delivery to Skin

**DOI:** 10.1002/adhm.71569

**Published:** 2026-09-09

**Authors:** Aydin Sadeqi, Gülçin Arslan Azizoğlu, Mark R. Prausnitz

**Affiliations:** ^1^ School of Chemical and Biomolecular Engineering Georgia Institute of Technology Atlanta Georgia USA

**Keywords:** biodegradable polymer, STAR particles, topical skin permeation, transdermal drug delivery

## Abstract

Drug delivery via skin enables local delivery that targets the skin and systemic delivery that avoids limitations of oral and parenteral administration. However, this route is often constrained by the skin's natural barrier, the stratum corneum. STAR particles with microscopic needles can painlessly puncture the skin to increase skin permeability, but they have previously been made of non‐biodegradable materials, such as ceramic and metal. To address potential environmental and safety concerns, we introduce biodegradable polymer STAR particles that are water‐soluble (poly(vinyl alcohol), PVA), enzyme‐degradable (cellulose acetate, CA), or hydrolyzable (polylactic acid, PLA). STAR particles were fabricated using femtosecond laser micromachining to achieve dimensional features and sharp tips. We characterized their mechanical properties, and confirmed their skin‐puncturing ability through gentian violet staining. We also evaluated STAR particle‐enhanced delivery of three model drugs in porcine skin *ex vivo*: tacrolimus with PVA STAR particles, methotrexate with CA STAR particles, and copper tripeptide‐1 with PLA STAR particles. Skin treatment with polymer STAR particles increased intradermal drug delivery by up to 37‐fold, depending on the drug type and the STAR particle material and application duration. We conclude that biodegradable polymer STAR particles can address environmental and safety concerns while enhancing drug delivery to skin.

## Graphical abstract

Biodegradable polymer STAR particles fabricated from water‐soluble, enzyme‐degradable, and hydrolyzable materials significantly enhance intradermal drug delivery. By replacing conventional non‐biodegradable materials, these STAR particles address environmental and safety concerns while expanding the potential of skin‐based drug delivery technologies.

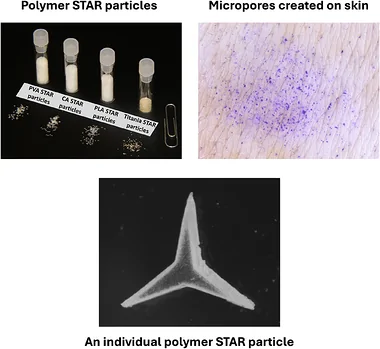

## 1 Introduction

Drug administration to the skin via topical delivery is an effective approach for treating dermatological conditions, as it allows direct targeting of the skin, enhancing efficacy and reducing systemic side effects. By delivering active drugs directly to the site of action, systemic exposure is minimized, improving safety and therapeutic outcomes [1, 2]. However, this method faces significant challenges. The outer layer to the skin, that is, stratum corneum, acts as a major barrier, limiting the delivery and thereby the effectiveness of most drugs [3, 4, 5, 6]. Various strategies have been proposed to overcome these barriers, which can be broadly categorized into formulation‐based approaches and physical methods.

Formulation‐based approaches typically use chemical penetration enhancers, which are commonly found in dermatological and cosmetic formulations to improve skin permeability [7]. While optimized formulations can significantly improve drug delivery to skin, their use is often limited by the need to avoid skin irritation and safety concerns. These issues restrict their broader application, emphasizing the need for careful formulation and consideration of alternative methods [5, 7].

Physical methods for increasing skin permeability include iontophoresis [8], thermal ablation [9], electroporation [10, 11], ultrasound [12], radiofrequency energy [13], microneedle patches [14, 15], and synergistic techniques [16] and thereby generally enable larger increases in skin permeability compared to formulation‐based approaches, including delivery of hydrophilic drugs and macromolecules like biologics. Despite their potential, these methods are often expensive, complex, and reliant on external power sources, limiting their practicality. Additionally, they are typically restricted to small areas of skin, further reducing their utility.

To address these concerns, we recently developed STAR particles as a novel approach to enhance skin permeability through the creation of micropores [17]. STAR particles were designed to have the advantages of formulation‐based approaches (i.e., low cost/complexity, simple self‐administration, applicable to large and variable areas of skin) and of physical methods (i.e., large increases in skin permeability to a broad variety of drug types). STAR particles accomplish this by having the functionality of microneedles, but administering them mounted on particles instead of via patches. This differs from typical microneedle patches, where the drug is incorporated into the microneedles or patch matrix, thereby limiting drug loading by the available patch volume. Instead, the drug is dissolved or dispersed in the topical formulation that carries the STAR particles. As a result, drug loading is primarily determined by the formulation concentration and applied volume rather than by the STAR particles themselves. The STAR particles are applied by gently rubbing them on the skin, where they painlessly puncture the stratum corneum to form micropores [18]. Drug delivery therefore occurs through enhanced drug diffusion via STAR‐generated micropores across the epidermis rather than by release from the STAR particles themselves.

STAR particles have shown significant potential in delivering dermatological drugs and macromolecules to the skin [17]. They can facilitate intradermal drug delivery for local skin treatment and transdermal drug delivery for systemic therapy. From an end‐user perspective, STAR particles are easy to use, integrating seamlessly into conventional topical formulations without the complexities of mechanical or physical methods like external power sources or specialized application techniques. Unlike most mechanically based methods, which are typically restricted to small skin areas, STAR particles can be applied to large regions of skin, defined in size and geometry by where the user rubs them. STAR particles are specifically designed with multiple arms to prevent embedding in the skin, thereby avoiding risks such as cutaneous granulomas. The safety, tolerability, and user acceptability of STAR particles treatment was recently demonstrated in human subjects [18].

The first generation of STAR particles have been fabricated out of metal (stainless steel) and ceramic (titania), which are generally considered biocompatible, but are not biodegradable [17]. The waste generated by these non‐degradable materials raises possible environmental and safety concerns. While chemical/material characteristics of these non‐biodegradable STAR particles are probably not of concern, their spiky shape could pose unknown risks to the environment. Furthermore, the possibility of STAR particles transferring to unintended tissues (e.g., eyes or gastrointestinal tract) or even to other individuals, raises possible safety concerns.

Here, we introduce second‐generation STAR particles that address these environmental and safety issues, by developing polymer STAR particles that biodegrade after use. We made STAR particles out of materials that are water‐soluble [19, 20, 21], enzyme‐degradable [22, 23], or hydrolyzable [24, 25, 26] using poly(vinyl alcohol) (PVA), cellulose acetate (CA), and polylactic acid (PLA), respectively. Unlike metal or ceramic STAR particles, STAR particles made of PVA, CA, or PLA degrade over time, providing a safer and more sustainable alternative.

To maintain drug delivery efficacy, biodegradable polymer STAR particles must retain the mechanical strength required to create micropores effectively while still addressing hypothetical concerns related to their unintended retention in skin that may lead to foreign body reactions and granuloma formation. Although metal and ceramic STAR particles are strong and have shown no evidence of breaking off in the skin, PVA, CA, and PLA STAR particles, being less mechanically robust, have a greater risk of fragmentation. However, their biocompatibility and biodegradability allow any fragments to safely degrade, dissolve and be cleared from the body [27, 28, 29, 30, 31, 32, 33, 34, 35], eliminating long‐term safety concerns. Likewise, material biodegradability enables polymer STAR particles that are introduced into the environment after use to similarly biodegrade, thereby eliminating their ability to puncture tissue upon STAR particle tip blunting and fully disappearing upon complete biodegradation.

Specifically, water‐soluble STAR particles should deactivate almost immediately upon puncturing the skin as their tips dissolve, while enzyme‐degradable and hydrolyzable STAR particles biodegrade more gradually, eventually weakening and disintegrating in the presence of abundantly present enzymes and water, respectively. From a use acceptability standpoint, polymer STAR particles offer the additional advantage of being translucent or transparent, making them less noticeable on the skin compared to the visible metal or ceramic STAR particles.

In this study, we characterized the size, shape, dimensions, and mechanical properties of the PVA, CA, and PLA STAR particles, demonstrating their ability to create micropores during application in porcine skin *ex vivo*. Additionally, we investigated the delivery of tacrolimus, methotrexate, and copper tripeptide‐1, selected for their relevance in treating inflammatory skin conditions, autoimmune diseases, and skin repair, using PVA, CA, and PLA STAR particles, respectively, again using porcine skin *ex vivo*. Combined, we believe that these studies showcase the potential of biodegradable polymer STAR particles as an effective and user‐friendly method of drug delivery to the skin.

## 2 Results

### 2.1 Design, Fabrication, and Characterization of STAR Particles

Our objective was to design STAR particles that address potential environmental and patient safety concerns, while retaining the drug delivery properties of current STAR particles to broadly increase skin delivery of drugs. Our overall strategy was to make STAR particles using materials that are biocompatible and biodegradable using three different classes of materials.

Our first approach involved making STAR particles using water‐soluble materials. When administered to the skin in a dry state, water‐soluble STAR particles can be strong and thereby puncture the stratum corneum, but upon contacting interstitial fluid in the skin, the STAR particles will dissolve and lose sharpness. The second approach was to use non‐water soluble, but biodegradable materials, that will break down into water‐soluble degradation byproducts through enzymatic degradation or hydrolysis. This second kind of STAR particles does not deactivate immediately—like water‐soluble STAR particles do—but they do deactivate and also can be included in topical formulations containing water (which is needed for solubilization and delivery of many drugs). We designed these STAR particles to biodegrade via enzymatic degradation by enzymes commonly found in nature [36, 37] and to biodegrade via hydrolysis by exposure water [38, 39].

Because STAR particles are designed to have the skin‐puncturing functionality of microneedles conventionally found in patches, we first considered materials used in microneedle patches as candidate materials for biodegradable STAR particles [40, 41, 42]. Within that context, we included a number of other considerations in our analysis. We first considered material type, focusing on materials that are biocompatible for safety issues and have sufficient mechanical strength to puncture skin. We also prioritized materials with low cost.

Considering compatibility with STAR particle fabrication, we prioritized materials suitable for manufacturing by laser micromachining, which we selected based on its ability to cut STAR particles with precisely controlled dimensions, as done in previous studies of STAR particle fabrication [17, 18, 43, 44]. Because laser micromachining cuts sheets of targeted materials, our choice of material needed to align with available sheet production methods, such as solution casting and hot‐pressing [45, 46, 47], and produce sheets that remained stable during exposure to environmental humidity and other conditions during handling. Our selected material also needed to be suitable for micromachining by laser cutting. For example, in prior studies [17, 18, 44], we utilized a CO_2_ laser to fabricate ceramic STAR particles. This method generates substantial heat during cutting, which can melt polymers, thereby deforming STAR particle shape and reducing microneedle tip sharpness. Therefore, our material selection and laser cutting parameters, needed to consider material heat tolerance during fabrication.

**FIGURE 1:**
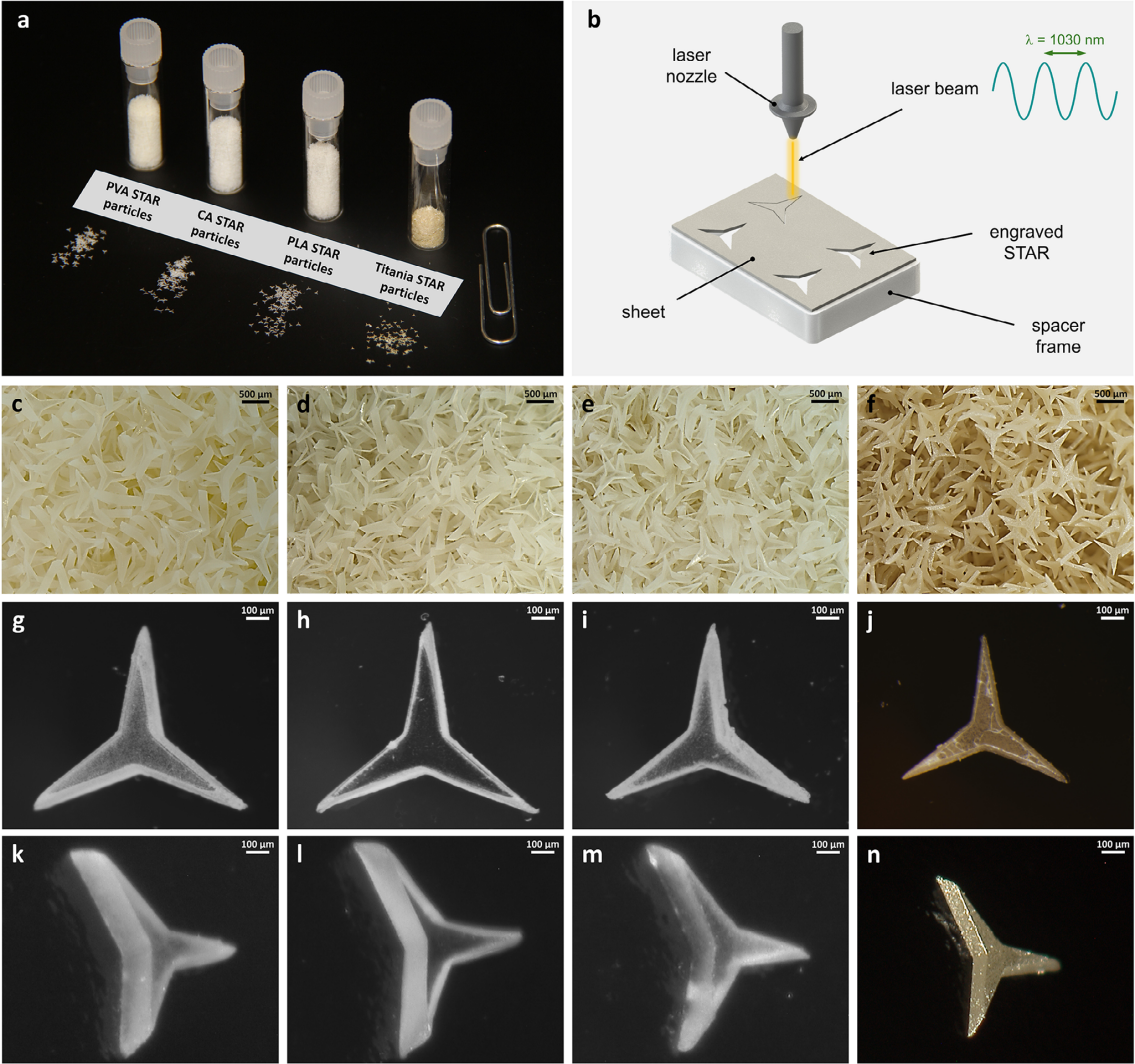
Water‐soluble, enzyme‐degradable, hydrolyzable, and non‐degradable STAR particles. (a) Representative images of PVA, CA, PLA, and titania STAR particles prepared as 200 mg batches. (b) STAR particle fabrication method using a femtosecond laser. Representative low‐magnification images of STAR particles made of (c) PVA, (d) CA, (e) PLA, and (f) titania. Representative high‐magnification views of individual STAR particles imaged from above – (g) PVA, (h) CA, (i) PLA, and (j) titania – and from the side – (k) PVA, (l) CA, (m) PLA, and (n) titania.

Based on these considerations, we selected PVA as a suitable material for water‐soluble STAR particles duo to its biocompatibility, cost‐effectiveness and widespread use in pharmaceutical formulations [48, 49, 50, 51, 52, 53, 54, 55]. PVA is also commonly used in microneedle patch fabrication, demonstrating strong mechanical properties for skin insertion [56]. Using a simple solution‐casting method, we were able to fabricate PVA sheets and cut STAR particle shapes from the sheets using a femtosecond laser operating at a near‐infrared wavelength of 1030 nm (Figure 1b). We chose this type of laser because its ultra‐short pulse length minimizes thermal effects that could melt the polymer, while cutting the PVA sheets by cold ablation through plasma formation [57, 58, 59]. We also added a spacer frame to prevent contact and adhesion between the PVA sheets and the cutting stage (Figure 1b). This approach created fine star‐shaped structures with sharp tips and a tapered profile, without significantly melting the PVA sheets (Figure 1c,g,k).

In initial trials, we also explored fabrication use other water‐soluble materials such as poly(vinyl pyrrolidone) (PVP) and isomalt. However, solution‐cast PVP sheets exhibited brittleness, posing challenges in handling and fabrication. Similarly, isomalt sheets, prepared using a hot press, showed high hygroscopicity and reduced durability under ambient conditions. Despite these challenges, we successfully fabricated PVP STAR particles and experimented with PVA/PVP blends, which reduced brittleness and improved handling of the sheets (Figure S1 in Supporting Information).

CA and PLA were identified as candidates for the categories of enzyme‐degradable and hydrolyzable STAR particles, as they also have been previously used in microneedle patch fabrication, supporting reliability for skin insertion [60, 61]. CA and PLA sheets were available for purchase commercially and remained stable at room temperature. We were able to cut STAR shapes with sharp tips and tapered structures on the sheets using the same femtosecond laser method developed for PVA STAR particles (Figure 1d,e,h,i,l,m).

Ceramic STAR particles made from titania sheets were fabricated as a positive control (Figure 1f,j,n), as they have been well‐explored in the literature [17, 18, 44]. When fabricating titania STAR particles, we had an additional sintering step to complete the fabrication process, as described previously [17, 18, 44].

Additional characterization showed that titania STAR particles exhibited size shrinkage due to the sintering process (Figure 1j,n). Side views of the STAR particles highlighted the tapered structure of the arms (Figure 1k–n), which was achieved by engraving three parallel traces of the STAR shapes, spaced closely apart (See Figure S2 in Supporting Information for more details). The sharper tapered tips were designed to enhance the STAR particles puncture into skin.

**FIGURE 2:**
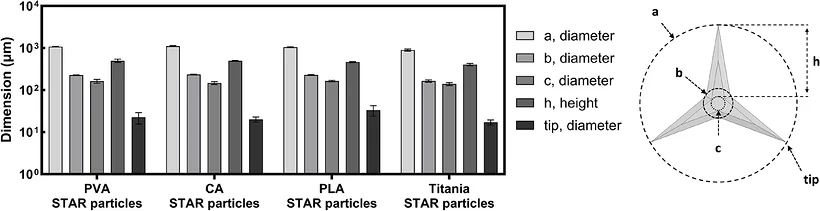
Dimensional characterization of PVA, CA, PLA, and titania STAR particles. Data are presented as mean ± standard deviation (*n* = 15 individual STAR particles per material). No inferential statistical comparisons were performed.

We next quantified the STAR particle dimensions, including the outer, middle, inner, and tip diameters, along with the microneedle arm heights (Figure 2, Table S1 in Supporting Information). Dimensional measurements are presented as mean ± standard deviation (SD), with *n* = 15 individual STAR particles analyzed per material. We found that PVA, CA, and PLA STAR particles had consistent dimensions, whereas titania STAR particles, due to the sintering‐induced shrinkage, exhibited smaller tip diameters, potentially offering improved skin penetration. These measurements were evaluated descriptively, and no inferential statistical comparisons were performed. The three types of polymer STAR particles all appeared translucent or transparent nature, making them less noticeable when left on the skin, whereas the titania STAR particles appeared yellow, making them more visible to the eye (Figure 1, Table S1 in Supporting Information).

### 2.2 Mechanical Properties of STAR Particles

Although PVA, CA, and PLA have been shown to be suitable materials for fabrication of microneedles in patches and mechanically reliable for skin insertion [48, 56, 60, 61], their performance as materials for STAR particles has not been previously evaluated. The different forces at play when applying STAR particles to the skin, including a combination of forces perpendicular and parallel to the skin surface, differ from those experienced by microneedle patches, which are applied normal perpendicular to the skin surface.

**FIGURE 3:**
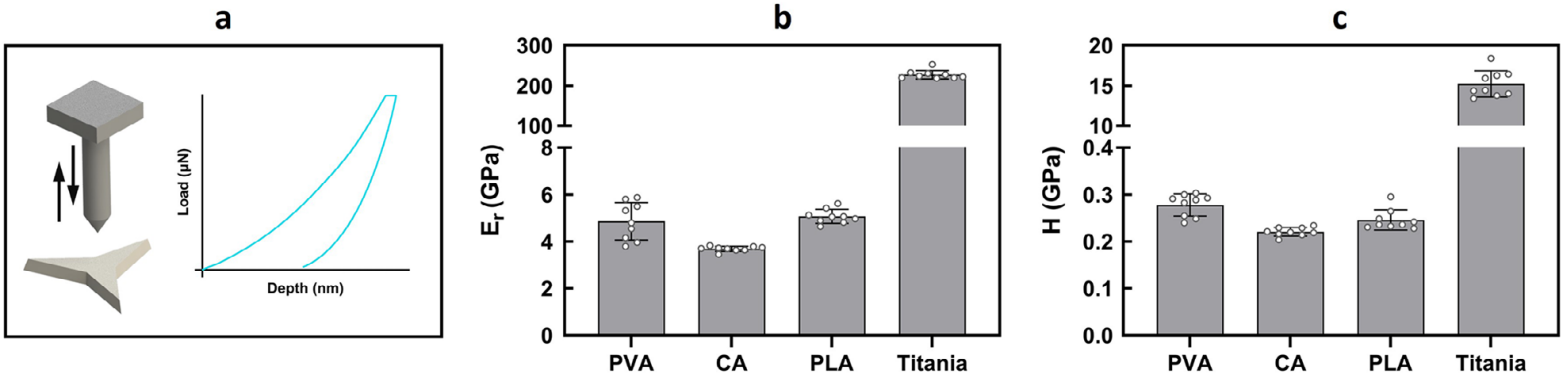
Mechanical properties of PVA, CA, PLA, and titania STAR particles. (a) Schematic concept of nano‐indentation. (b) Reduced modulus and (c) hardness of PVA, CA, PLA, and titania STAR particles. Data are presented as mean ± standard deviation, and individual data points are shown (*n* = 9 independently prepared disks per material). Statistical differences were assessed using Welch's one‐way ANOVA followed by Dunnett's T3 post hoc multiple‐comparisons test, with titania as the control. Pairwise comparisons were two‐sided, and adjusted p‐values are reported. PVA, CA, and PLA differed significantly from titania for both reduced modulus and hardness (adjusted *p* < 0.0001 for each polymer‐versus‐titania comparison).

To address this, we conducted a comparative analysis of the mechanical properties of disks made of PVA, CA, or PLA against those made of titania, which served as a positive control. We used a nano‐indenter to calculate the reduced modulus and hardness from measurements of load versus depth of indentation (Figure 3a). The reduced modulus represents the combined elastic properties of two materials in contact, providing a measure of the effective stiffness of the system by incorporating elastic moduli and Poisson's ratios of both the indenter and the test material [62].

For both reduced modulus and hardness, data are presented as mean ± SD (*n* = 9 independently prepared disks per material). The reduced modulus values of PVA, CA, and PLA samples were significantly lower than that of titania (Welch's one‐way ANOVA followed by Dunnett's T3 post hoc multiple‐comparisons test; adjusted *p* < 0.0001 for each polymer‐versus‐titania comparison), consistent with the higher stiffness expected for a ceramic material (Figure 3b). Similarly, hardness measurements showed that PVA, CA, and PLA exhibited significantly lower values than titania (Welch's one‐way ANOVA followed by Dunnett's T3 post hoc multiple‐comparisons test; adjusted *p* < 0.0001 for each polymer‐versus‐titania comparison; Figure 3c).

### 2.3 Skin Puncture Performance of STAR Particles

Because the mechanical properties of PVA, CA, and PLA revealed significantly lower reduced modulus and hardness compared to titania STAR particles, we assessed the ability of polymer STAR particles to puncture skin using both freshly made STAR particle formulations and STAR‐particle formulations stored for one week.

#### 2.3.1 PVA STAR Particles

**FIGURE 4:**
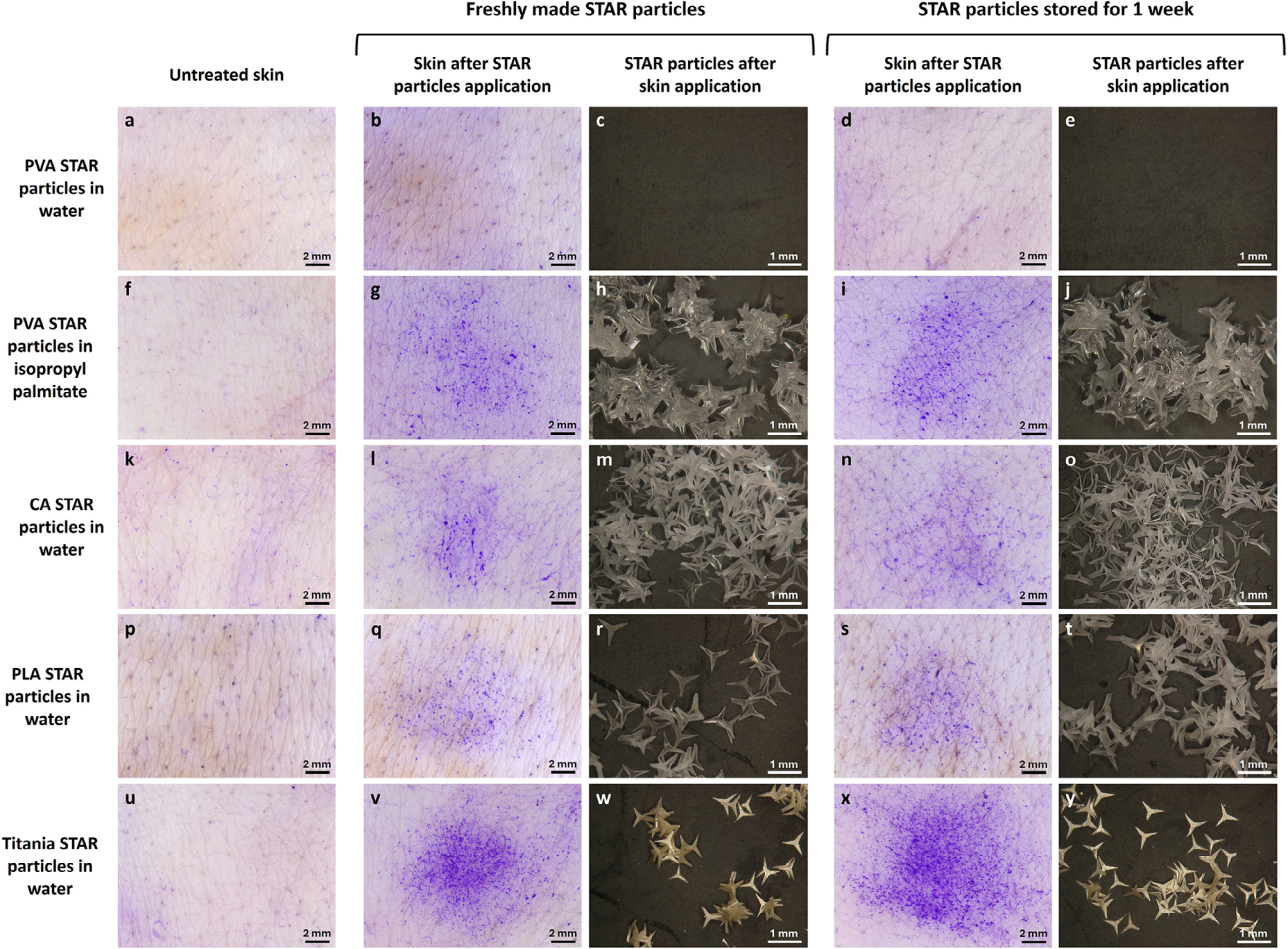
Skin puncture performance of STAR particles freshly made and after one week of storage in aqueous or non‐aqueous formulations. Representative images of the skin surface before application of STAR particles (a, f, k, p, u), after application of STAR particles in freshly made formulations (b, g, l, q, v), and after application of STAR particles in formulations stored for one week (d, i, n, s, x). In each case, gentian violet staining of skin puncture sites was performed immediately after STAR particle treatment. Representative images of STAR particles in freshly made formulations after skin application (c, h, m, r, w) and in formulations stored for one week after skin application (e, j, o, t, y). Images show: untreated skin (a, f, k, p, u), results from STAR particles made of PVA in a water vehicle (b, c, d, e), STAR particles made of PVA in an isopropyl palmitate vehicle (g, h, i, j), STAR particles made of CA in a water vehicle (l, m, n, o), STAR particles made of PLA in a water vehicle (q, r, s, t), and STAR particles made of titania in a water vehicle (v, w, x, y). STAR particles were prepared at a concentration of 10% w/v) and rubbed on porcine skin *ex vivo* for 10 s. Week‐old STAR particles were stored in their delivery vehicle at room temperature for one week.

PVA STAR particles were first suspended in water‐based aloe vera gel and tested for their skin‐puncturing ability. As expected, no skin puncture was observed due to the rapid dissolution of the PVA STAR particles in the aqueous vehicle (Figure 4a–e). This confirms that PVA STAR particles are unsuitable for water‐based vehicles. PVA STAR particles were therefore suspended in various non‐aqueous vehicles, including mineral oil, olive oil, propylene glycol, isopropyl palmitate, isopropyl myristate and polyethylene glycol (Table S2 in Supporting Information). When suspended in any of these non‐aqueous vehicles, STAR particles in freshly made formulations were able to puncture skin, and STAR particles in any of the formulations other than propylene glycol were also able to puncture skin. STAR particles stored in propylene glycol became soft, which made them ineffective for skin puncture.

Using PVA STAR particles suspended in isopropyl palmitate as a non‐aqueous vehicle, we found that both fresh and week‐old formulations successfully punctured the skin with similar performance (Figure 4f–j). Post‐application microscopic imaging (Figure 4h,j) confirmed that the STAR particles remained intact, showing no signs of damage after use. Isopropyl palmitate, widely used in dermatological formulations and cosmetics, is a non‐toxic ingredient that is considered safe when used in appropriate formulations [63].

#### 2.3.2 CA STAR Particles

CA STAR particles in water, using both fresh and week‐old formulations, demonstrated consistent skin‐puncturing ability (Figure 4k–o). The retrieved STAR particles after application showed no visible damage, indicating durability and suitability for use in water‐based vehicles (Figure 4m,o).

#### 2.3.3 PLA STAR Particles

PLA STAR particles in water, whether fresh or stored for one week, effectively punctured the skin (Figure 4p–t). The retrieved STAR particles showed no signs of damage, confirming their robustness during application (Figure 4r,t). However, we expected that extended storage of PLA STAR particles in water will lead to degradation by hydrolysis [24, 25, 26], thereby weakening the STAR particles over time and limited the shelf life of PLA STAR particles in aqueous formulations. Therefore, PLA STAR particles might need to be reconstituted in water shortly before use or prepared in a non‐aqueous formulation using a solvent in which PLA is not soluble.

#### 2.3.4 Titania STAR Particles

Fresh and week‐old titania STAR particles in water displayed consistent puncturing performance (Figure 4u–y), generating a greater number of pores compared to the PVA, CA, and PLA STAR particles, according to qualitative visual inspection. This difference is likely due to the higher modulus and hardness of titania STAR particles, allowing them to maintain close contact with the skin and puncture more effectively with each rubbing motion on the skin. Post‐application imaging of titania STAR particles revealed some broken microneedle arms, likely due to the material's brittleness (Figure 4w,y).

### 2.4 Delivery of Tacrolimus, Methotrexate, and Copper Tripeptide‐1 With STAR Particles

To assess the ability and versatility of polymer STAR particles to deliver drugs to the skin, we paired each type of STAR particle—water‐soluble PVA, enzyme‐degradable CA, and hydrolysable PLA—with a different drug—tacrolimus, methotrexate, and copper tripeptide‐1, respectively—and measured intradermal drug delivery in porcine skin *ex vivo*. Although our primary interest in using these drugs was to evaluate local intradermal delivery for the treatment of skin, we also reported their transdermal delivery as a surrogate measure of systemic drug delivery.

#### 2.4.1 Tacrolimus Delivery Using PVA STAR Particles

Since PVA STAR particles require a non‐aqueous vehicle, they are best suited for drugs soluble in non‐aqueous solvents. We selected tacrolimus as the model drug for delivery using PVA STAR particles. Tacrolimus is used to treat inflammatory skin conditions like atopic dermatitis [64], but has limited skin permeability due to its molecular weight (804 g/mol) despite relatively high lipophilicity (XLogP3 = 2.7) [65]. Tacrolimus has limited water solubility (4–12 µg/mL) but is soluble in non‐aqueous solvents such as isopropyl palmitate [65, 66, 67].

**FIGURE 5:**
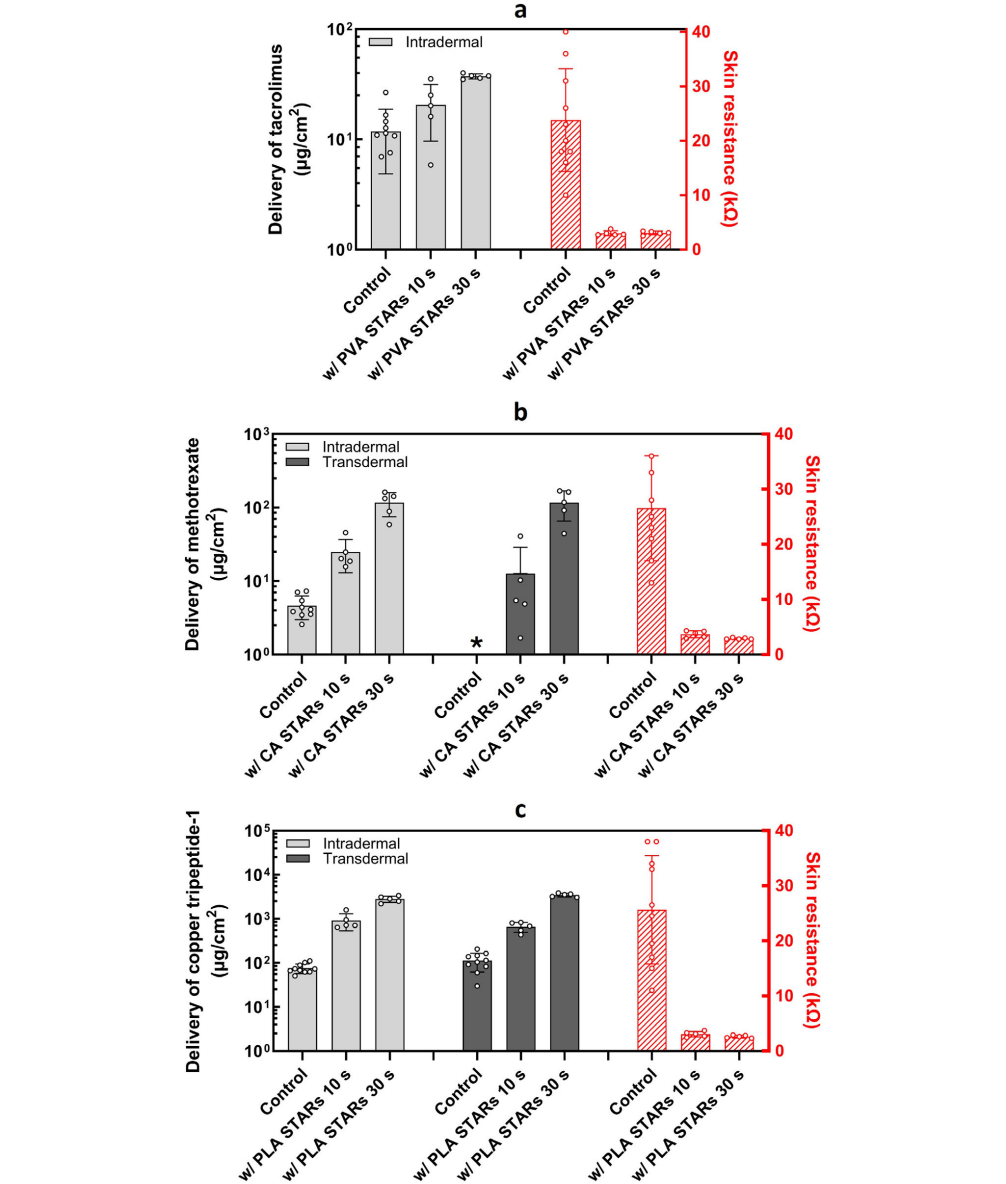
Delivery of tacrolimus, methotrexate, and copper tripeptide‐1 using PVA, CA, and PLA STAR particles, respectively. STAR particles were rubbed onto *ex vivo* porcine skin for 10 or 30 s. Intradermal and transdermal drug delivery were assessed 24 h after treatment, and skin electrical resistance was measured after skin hydration. (a) Intradermal delivery of tacrolimus and electrical resistance of skin after PVA STAR particle treatment using a formulation of 0.1% (w/v) tacrolimus and 10% (w/v) PVA STAR particles in isopropyl palmitate (For both intradermal delivery and skin electrical resistance, *n* = 10 independent porcine skin samples for the control group; *n* = 5 independent porcine skin samples for each 10 and 30 s PVA STAR particle‐treated group). Compared with the control, intradermal tacrolimus delivery after 10 and 30 s of treatment had adjusted *p* = 0.27 and adjusted *p* < 0.0001, respectively, whereas skin electrical resistance was reduced after both treatment durations (adjusted *p* < 0.05 for each comparison). Transdermal tacrolimus was below the detection limit in all groups and is not shown. (b) Intradermal and transdermal delivery of methotrexate and electrical resistance of skin after CA STAR particle treatment using a formulation of 0.1% (w/v) methotrexate and 10% (w/v) CA STAR particles in water (For intradermal and transdermal delivery and skin electrical resistance, *n* = 9 independent porcine skin samples for the control group; *n* = 5 independent porcine skin samples for each 10 and 30 s CA STAR particle‐treated group). Compared with the control, intradermal methotrexate delivery and skin electrical resistance differed after both treatment durations (adjusted *p* < 0.05 for each comparison), whereas transdermal delivery differed after both treatment durations (adjusted *p* < 0.0001 for each comparison). The asterisk (*) indicates that the transdermal methotrexate in the control group was below the detection limit. (c) Intradermal and transdermal delivery of copper tripeptide‐1 and electrical resistance of skin after PLA STAR particle treatment using a formulation of 5% (w/v) copper tripeptide‐1 and 10% (w/v) PLA STAR particles in water. Intradermal and transdermal drug delivery were assessed 24 h after STAR particle treatment (For intradermal and transdermal delivery and skin electrical resistance, *n* = 10 independent porcine skin samples for the control group; *n* = 5 independent porcine skin samples for each 10 and 30 s PLA STAR particle‐treated group. Compared with the control, intradermal and transdermal copper tripeptide‐1 delivery increased, and skin electrical resistance decreased after both treatment durations (adjusted *p* < 0.05 for each comparison). Each porcine skin sample was tested in a separate Franz cell and constituted one independent experimental replicate. Data are presented as mean ± standard deviation, and individual data points are shown. Each outcome was analyzed separately using Welch's one‐way ANOVA followed by two‐sided Dunnett's T3 post hoc multiple‐comparisons testing, with the corresponding untreated group as the control. Adjusted *p*‐values are reported.

Skin treatment with PVA STAR particles in isopropyl palmitate was effective to reduce skin barrier properties, as shown by a drop in skin electrical resistance from 23.8 kΩ in control samples to 2.7 kΩ or 2.5 kΩ after rubbing PVA STAR particles on the skin for 10 or 30 s, respectively (data are presented as mean ± SD; n = 10 independent porcine skin samples for the control group and n = 5 independent porcine skin samples for each treated group; Welch's one‐way ANOVA followed by two‐sided Dunnett's T3 post hoc multiple‐comparisons testing; adjusted *p* < 0.05 for each comparison with control; Figure 5a). This drop in resistance confirms micropore formation, which is expected to correspond to enhanced drug permeability.

After topical tacrolimus delivery for 24 h, rubbing the skin with PVA STAR particles for 10 s or 30 s produced a 1.7‐ or 3.2‐fold increase (adjusted *p* = 0.27 for 10 s rubbing; adjusted *p* < 0.0001 for 30 s rubbing) in intradermal delivery of tacrolimus (i.e., drug measured within the skin), respectively, compared with control samples without STAR particle treatment (Figure 5a). However, transdermal delivery (i.e., drug measured in the Franz cell receptor chamber after fully crossing the skin) was undetectable in both control and STAR particle‐treated samples, probably due to the low solubility of tacrolimus in the phosphate‐buffered saline (PBS) solution in the receptor chamber.

#### 2.4.2 Methotrexate Delivery Using CA STAR Particles

Because we found that CA STAR particles are compatible with aqueous vehicles, we selected methotrexate as the model drug for delivery using CA STAR particles. Methotrexate is commonly prescribed for psoriasis treatment, but is administered systemically with significant side effects [68], since methotrexate cannot be given topically to the skin due to its very low cutaneous permeability caused by its relatively high molecular weight (454 g/mol) and strong hydrophilicity (XLogP3 = −1.8) [69].

Electrical resistance measurements confirmed skin puncturing by CA STAR particles, dropping from 26.6 kΩ to 3.7 kΩ or 2.9 kΩ after 10 or 30 s of CA STAR particles application to the skin, respectively (data are presented as mean ± SD; *n* = 9 independent porcine skin samples for the control group and *n* = 5 independent porcine skin samples for each treated group; Welch's one‐way ANOVA followed by two‐sided Dunnett's T3 post hoc multiple‐comparisons testing; adjusted *p* < 0.05 for each comparison with control; Figure 5b). Methotrexate delivery also demonstrated substantial improvements after CA STAR particle treatment, with intradermal delivery increasing 5.4‐ or 25.2‐fold after 10 or 30 s of application, respectively (adjusted *p* < 0.05 for each comparison with control; Figure 5b). Additionally, while transdermally delivered methotrexate was below the detection limit of 0.06 ± 0.03 µg/cm^2^ in control samples, it was significantly higher than the detection limit in the STAR particle‐treated samples (adjusted *p* < 0.0001 for each comparison; Figure 5b).

#### 2.4.3 Copper Tripeptide‐1 Delivery Using PLA STAR Particles

For skin delivery assessment, PLA STAR particles were paired with copper tripeptide‐1, a widely used skincare ingredient known for its anti‐aging, wound healing, and skin regeneration properties [70]. Copper tripeptide‐1 (402 g/mol) consists of the amino acids glycine, histidine, and lysine bound to copper and is believed to facilitate collagen synthesis and cellular transport [71].

Skin electrical resistance measurements showed a decrease from 25.7 kΩ to 3.1 kΩ or 2.6 kΩ after 10 or 30 s of PLA STAR particle application, respectively (data are presented as mean ± SD; n = 10 independent porcine skin samples for the control group and n = 5 independent porcine skin samples for each treated group; Welch's one‐way ANOVA followed by two‐sided Dunnett's T3 post hoc multiple‐comparisons testing; adjusted *p* < 0.05 for each comparison with control; Figure 5c). The delivery of copper tripeptide‐1 was significantly enhanced by PLA STAR particles, with intradermal delivery increasing by 12.1‐ or 37‐fold after 10 or 30 s of application, respectively (adjusted *p* < 0.05 for each comparison with control; Figure 5c). Transdermal delivery also improved under the same conditions (adjusted *p* < 0.05 for each comparison with control; Figure 5c).

## 3 Discussion

STAR particles have the potential to broadly increase the range of drug types that can be effectively administered through the skin, especially for local skin delivery to treat dermatological indications, but also for other local and systemic purposes [17, 43, 44]. This is because STAR particles create micropores in the skin across the stratum corneum that enable delivery of hydrophilic drugs and macromolecules, which are normally excluded from skin delivery by the stratum corneum barrier. Prior studies have shown enhanced delivery of 5‐fluorouricil, methotrexate, bleomycin, minoxidil, acyclovir, and tetanus toxoid in animal models [17, 18, 44] and a formulation of lidocaine/epinephrine/tetracaine in human subjects [43].

While prior studies have focused largely on ceramic STAR particles, this study introduced polymeric STAR particles made of water‐soluble and biodegradable materials, and demonstrated the ability of these STAR particles to be formulated in aqueous and non‐aqueous vehicles to effectively puncture the skin and increase intradermal and transdermal drug delivery. Development of these polymer STAR particles was motivated by the need to address possible safety, environmental, and aesthetic concerns with non‐degradable STAR particles made of ceramic, metal or other materials. As discussed below, the existence or significance of these concerns is unknown, but the use of water‐soluble and biodegradable STAR particles at a minimum reduces the need to study possible adverse effects and, better still, may eliminate them as a concern altogether. Our previous human studies with ceramic STAR particles demonstrated good skin tolerability, even after daily application for ten consecutive days [18]. We expect that the biodegradable STAR particles introduced in this study should be similarly well tolerated, this expectation should be addressed in future studies.

While studies of the effects of STAR particles on tissues other than skin in the context of application by forceful and directed rubbing has not yet been studied, there is a hypothetical concern that STAR particles could unintentionally end up in the eye, gastrointestinal tract or other part of the body of a user or of someone else after their initial use. If STAR particles retain sharp and strong microneedle tips, they could puncture these tissues, possibly leading to adverse effects. While a hypothetical risk, we believe the true risk to be small, since STAR particles must be forcefully applied to the skin to be effective. It is unclear that, for example, ocular blinking, intestinal motility, or other forces that might be unintentionally applied to STAR particles would have the right directionality and force to cause STAR particles to puncture tissue. Future studies are needed to assess these hypothetical risks, as well as more‐fully characterize the mechanical properties of STAR particles before and during their biodegradation.

These hypothetical risks, however, are essentially eliminated with water‐soluble STAR particles that should rapidly become deactivated upon contacting wet tissues, since their microneedle tips should almost immediately become blunt and the whole STAR particle should rapidly soften and then disappear by dissolution. Biodegradable STAR particles will likely retain their sharp tips and mechanical integrity immediately after use, but will eventually become blunt and weak upon enzymatic degradation or hydrolysis.

The environmental consequences of non‐degradable STAR particles discarded as solid waste, in the sewage system, or otherwise into the environment are also unknown and pose a hypothetical risk. While widely used materials like titania, alumina, stainless steel, and other materials should pose little or no chemical/material risk to the environment [72, 73, 74, 75, 76, 77, 78], a sharp STAR particle made of these materials might be of concern. Again, making STAR particles from water‐soluble and biodegradable materials like PVA, CA, and PLA reduces or eliminates possible risk to the environment, whether perceived or real.

Most topical formulations do not leave visible residue on the skin, even if they are initially visible upon application. STAR particles that have color or are otherwise not translucent or transparent, and do not quickly dissolve or degrade, may be visible on the skin, which could be of aesthetic concern if not washed off or otherwise removed. Water‐soluble STAR particles that may be visible upon application, can quickly dissolve and become invisible, as seen for PVA STAR particles. STAR particles made of other polymers, such as CA and PLA, may not dissolve right away, but if they are transparent or translucent, they may still have minimal distortion of skin appearance and therefore be aesthetically acceptable to users.

From a manufacturing standpoint, ceramic STAR particles, such as those made from titania, can be fabricated using high‐power, high‐speed CO_2_ laser micromachining, allowing rapid mass production with minimal operator intervention. Additionally, CO_2_ lasers are cost‐effective and in widespread use in pharmaceutical and other manufacturing processes for high‐precision cutting, engraving, marking, and welding, which will contribute to lower manufacturing costs [79]. In contrast, fabrication of polymer STAR particles, such as those made in this study with PVA, CA, and PLA, required the use of femtosecond lasers to prevent polymer melting, which are more expensive and have slower throughput [80]. This will likely contribute to increased production costs. An advantage, however, of making STAR particles from polymer is that it avoids the post‐laser‐cutting sintering step needed for ceramic STAR particles [17, 18, 44], which adds to production time and cost. These issues, however, may be eliminated by making STAR particles by other fabrication methods such as molding, which is already widely used in microneedle patch fabrication in advanced commercial development [81, 82]. While the present study demonstrates the feasibility of fabricating biodegradable polymer STAR particles using femtosecond laser micromachining, future studies should evaluate batch‐to‐batch reproducibility, manufacturing yield, throughput, and scalability of the fabrication process to assess commercial viability.

A limitation of polymer STAR particles is that they can only be used with vehicles that do not dissolve, degrade or soften the STAR particles, and are also biocompatible and sufficiently dissolve drugs of interest. Since aqueous vehicles are commonly used in topical formulations [83], this poses an especially significant limitation on water‐soluble STAR particles that must use non‐aqueous vehicles, and on STAR particles that degrade by hydrolysis, that have limited long‐term storage stability due to hydrolytic degradation. Enzymatically degradable STAR particles are more versatile, limited less by potential degradation than by material softening by vehicle penetration into the STAR particle, making it mechanically weak. Adverse interactions between STAR particles and their vehicles can be overcome by packaging the STAR particles and drug solution separately, requiring the patient to mix them before application. This approach, however, introduces risks of user non‐adherence and reduced user acceptability.

## 4 Conclusion

In this study, we introduced polymer STAR particles designed to be water‐soluble, enzyme‐degradable, or hydrolysable by making them out of PVA, CA, or PLA, respectively. To ensure precise fabrication and avoid heat absorption and melting by the polymer sheets, we fabricated the STAR particles using a femtosecond laser operating at a near‐infrared wavelength of 1030 nm, which produced STAR particles with sharp, well‐defined microneedle tips and a tapered profile. The dimensional features of the STAR particles were similar among all three types of polymer STAR particles. While their mechanical properties, including reduced modulus and hardness, were reduced relative to ceramic (titania) STAR particles, PVA, CA, and PLA STAR particles were nonetheless able to generate micropores in porcine skin *ex vivo*, even after storage in aqueous or non‐aqueous vehicles for one week.

The three types of polymer STAR particles were also able to increase intradermal and transdermal delivery of three different model drugs, as shown in porcine skin *ex vivo*. Tacrolimus formulated with PVA STAR particles is isopropyl palmitate exhibited intradermal delivery increased up to 3.2‐fold compared to delivery without STAR particles. Methotrexate formulated with CA STAR particles in water had intradermal delivery improved by up to 25.‐fold. Finally, copper tripeptide‐1 formulated with PLA STAR particles in water showed intradermal delivery increased by up to 37‐fold. These results highlight the versatility of STAR particles to enhance drug delivery to the skin with dramatically increase rates that depended on drug type and STAR particle material and application duration.

Overall, water‐soluble and biodegradable polymer STAR particles present a promising advancement in intradermal and transdermal drug delivery. Their deactivation and elimination by various mechanisms addresses possible concerns related to safety, environmental impact, and aesthetic acceptability. In this way, water‐soluble, enzyme‐degradable and hydrolizable STAR particles offer a safer and more sustainable alternative to existing technologies, paving the way for improved patient outcomes and broader applications.

## 5 Methods

### 5.1 Preparation of PVA, CA, PLA, and Titania Sheets

PVA sheets were made in‐house by casting a 10% (w/v) solution of PVA (Millipore Sigma, Burlington, MA, USA) in PBS (Corning, Manassas, VA, USA) on a mold made of EcoFlex 00–50 (Smooth‐On, Macungie, PA, USA). The molds contained cavities measuring 40×40×2 mm and were placed on a leveled hot plate at 40°C in a fume hood for ∼24 h. Cellulose acetate sheets were purchased from McMaster–Carr (Douglasville, GA, USA). PLA sheets were purchased from Virtues Culture (Shenzhen, China). Titania (TiO_2_) sheets were purchased from Maryland Ceramic & Steatite (Bel Air, MD, USA).

### 5.2 Fabrication of PVA, CA, PLA, and Titania STAR Particles

STAR particle shape was designed in AutoCAD (Autodesk, San Francisco, CA, USA). A femtosecond laser from Optec Laser Systems (San Diego, CA, USA) with a wavelength of 1030 nm was used to cut the STAR particles out of the sheets. To avoid contact between STAR particles and the cutting stage, the sheets were placed on a polymethyl methacrylate (PMMA) frame (McMaster‐Carr) while cutting the STAR particles. STAR particles were stored with desiccant at room temperature (20–25°C) until use. There was an additional step of sintering titania STAR particles after laser cutting, as described previously [17, 44].

### 5.3 Microscopic Imaging of STAR Particles

STAR particles were imaged using a Hirox digital microscope KH‐8700 (Hirox, Tokyo, Japan).

### 5.4 Measurement of Reduced Modulus and Hardness of PVA, CA, PLA, and Titania STAR Particles

Nano‐indentation is a widely used technique for determining the mechanical properties of materials and thin films [84, 85, 86]. This method involves applying a probe with known properties to a material with unknown properties under controlled force. Nano‐indentation was performed on disks (diameter of 2 mm; 120 µm thickness for PVA, CA, and PLA disks; 80 µm thickness for titania disks) using a Hysitron TI‐980 Triboindenter (Bruker, Billerica, MA, USA). A Berkovich tip (TI‐0039, Bruker), which is a three‐sided pyramid tip, was used for indentation, as it is the standard for nano‐indentation of thin films greater than 100 nm thick [84, 85, 86]. The reduced modulus (*E*_r_), which includes contributions from both the specimen and the indenter, was calculated by:

(1) $$\frac{1}{E_{r}}={\left(\frac{1-\;\nu^{\;2}}{E}\right)}_{\mathrm{specimen}}+{\left(\frac{1-\;\nu^{\;2}}{E}\right)}_{\mathrm{indenter}}$$

where *E* and *ν* are the elastic modulus and Poisson's ratio, respectively, of the specimen and the indenter.

The hardness was defined as:

(2) $$H=\frac{P_{\max}}{A}\;$$

where *P*_max_ is the maximum indentation force and *A* as the projected contact area at that load.

### 5.5 Skin Puncture Test

Freshly harvested porcine ears from pigs of mixed breeds and sexes, 6–9 months of age, were purchased postmortem from Pel‐Freez Biologicals (Rogers, AR, USA), and the skin was separated from the cartilage beneath. No live animals were used specifically for this study; therefore, institutional animal ethics approval was not required. Excess hair was shaved off with a disposable razor (Dynarex, Montvale, NJ, USA). The prepared ear skin was then rinsed with water at room temperature, wrapped in aluminum foil, and stored at ─80 °C until use. Skin samples with visible defects, such as cuts or tears in the stratum corneum, were excluded from experiments.

Prior to the skin puncture test, pig skin samples were thawed at room temperature and then hydrated in a PBS solution containing 10 mM sodium azide (PBS/azide, with sodium azide from Sigma–Aldrich, St. Louis, MO, USA) for ∼8 h. After that, the skin was placed on a flat surface and a gentian violet solution (Humco, Texarkana, TX, USA) was applied to stain the skin, ensuring complete coverage of the entire surface. After 10 min, the gentian violet was removed using paper towels (Kimwipe, Kimberly–Clark, Roswell, GA, USA) followed by 70% isopropyl alcohol swabs (BD Alcohol Swabs, Becton Dickinson, Franklin Lakes, NJ, USA). Skin samples with visual defects, including cuts or holes in the stratum corneum indicated by gentian violet staining, were discarded following thorough visual inspection. The prepared skin samples were imaged using light microscopy with a SZX16 microscope (Olympus, Tokyo, Japan) with a DP71 camera (Olympus).

STAR particles were incorporated at a concentration of 10% (w/v) in an aqueous or non‐aqueous vehicle. The STAR particle formulation was applied to the skin by circular rubbing with a finger for 10 s, applying a force of approximately 5–15 N over an area of ∼20 cm^2^. No evaporation of the liquid formulation vehicle was noted during the brief STAR particle application. The hands of the investigator were covered with double nitrile gloves (Microflex Powder‐Free Nitrile gloves, Avantor, Radnor, PA, USA) for protection during application. Following application, the STAR particles were removed from the skin using alcohol swabs. The skin was then re‐stained with gentian violet to identify puncture sites created by the STAR particles. After 10 min, the gentian violet was wiped off with kimwipes and alcohol swabs. Finally, the treated areas were imaged using a SZX16 microscope with a DP71 camera.

### 5.6 Intradermal and Transdermal Drug Delivery Using STAR particles

The skin preparation and STAR particles application were performed as described for the Skin Puncture Test. After removing the STAR particles from the skin, the treated skin areas were cut into circular samples with a diameter of ∼24 mm and loaded into Franz cells (PermeGear, Riegelsville, PA, USA) in which the exposed skin diameter was ∼9 mm. Untreated skin areas without visible pores or cuts were selected as control samples.

The receptor chamber of the Franz cell was filled with PBS/azide. The donor chamber was sealed with parafilm to prevent leakage and evaporation. The donor chamber was initially filled with PBS/azide, and the cell was refrigerated for ∼8 h to allow hydration of the skin. Electrical resistance of the skin was measured at this stage, as described below. Following hydration, the donor chamber was emptied and refilled with 1 mL of drug solution, prepared according to the concentrations and solvents listed in Table 1. The Franz cells were maintained at a constant temperature of 37°C with stirring at ∼300 rpm using a PermeGear HS‐2 system. After 24 h, skin samples were taken out of the Franz cells, stratum corneum removed with skin stripping adhesive discs (Clinical and Derm, Dallas, TX, USA), cut into nine pieces of approximately equal size, and submerged in the drug extraction solution specified in Table 1. The extraction process was carried out for ∼24 h at room temperature under continuous stirring at ∼1000 rpm.

**TABLE 1:** Drug formulations and extraction solvents used in drug delivery studies.

Drug	Solvent	Drug extraction solution
0.1% Tacrolimus	Isopropyl palmitate	Acetone
0.1% Methotrexate	NaOH (10 mM) in DI water (pH 11.5)	NaOH (10 mM):methanol (1:1 v/v)
5% Copper tripeptide‐1	DI water	0.1% trifluoroacetic acid in DI water

Drug concentrations in the Franz‐cell receptor solution and skin‐extraction solution were quantified by HPLC. Transdermal drug delivery was calculated by multiplying the drug concentration measured in the receptor solution (µg/mL) by the receptor‐chamber volume (mL) and dividing the resulting drug amount by the exposed skin area (cm^2^). Intradermal drug delivery was calculated by multiplying the drug concentration measured in the skin‐extraction solution (µg/mL) by the extraction‐solution volume (mL) and dividing the resulting drug amount by the exposed skin area (cm^2^). The exposed skin area was calculated from the Franz‐cell opening diameter using A = π (d/2)^2^. Intradermal and transdermal delivery values were reported in µg/cm^2^.

For these studies, tacrolimus monohydrate and acetone were purchased from Sigma–Aldrich. Methotrexate and isopropyl palmitate, were purchased from TCI (Portland, OR, USA). Sodium hydroxide and methanol were purchased from Avantor. Copper tripeptide‐1 powder was purchased from Dermafactors (Frostproof, FL, USA). Trifluoroacetic acid was purchased from Millipore (Billerica, MA, USA).

### 5.7 Measurement of Skin Electrical Resistance

Electrical resistance was measured using a Fluke 117 digital multimeter (Fluke, Everett, Washington, USA). Ag/AgCl electrodes (World Precision Instruments, Sarasota, FL, USA) were submerged in the donor chamber and receiver chamber sampling port to measure the resistance between two sides of the skin. The measurements were done after skin hydration when both donor and receiver chambers were filled with PBS/azide.

### 5.8 HPLC Methods

An HPLC system (Agilent 1260 Infinity II LC System, Agilent, Santa Clara, CA, USA), equipped with a quaternary pump, autosampler, and diode array detector, was employed to quantify the delivery of drug into and across porcine skin *ex vivo*. For analysis of tacrolimus and methotrexate, a C18 column (Zorbax Eclipse XDB‐C18; 3.5 µm particle size, 4.6 mm inner diameter × 150 mm length, Agilent) was used. Copper tripeptide‐1 was analyzed with an Aeris peptide column (XB‐C18 column, 3.6 µm particle size, 4.6 mm inner diameter × 250 mm length). The operating parameters used for quantification are presented in Table 2. All mobile phase solvents were HPLC grade (Sigma–Aldrich). The flow rate of mobile phase was set at 1 mL/min and an injection volume of 10 µL was used throughout all three studies.

**TABLE 2:** Summary of HPLC operating parameters for quantification of skin delivery ex vivo.^a^

Drug	Mobile phase	T (°C)	*λ* (nm)	RT (min)	Method time (min)
Tacrolimus	ACN:water (65:35)	60	215	9.58	13
Methotrexate	ACN:0.1% TFA in water (15:85)	30	303	4.8	10
Copper tripeptide‐1	0.1% TFA in DI water	Not controlled	229	3.8	10

^a^ Abbreviations: ACN, acetonitrile; RT, retention time; T, column temperature; TFA, trifluoroacetic acid; λ, UV detection wavelength.

### 5.9 Statistical Analysis

Statistical analyses were performed using GraphPad Prism 10 (Boston, MA, USA). Before statistical analysis, HPLC‐derived drug concentrations were converted to the delivered drug amount per unit skin area (µg/cm^2^) using the corresponding receptor or extraction volume and exposed skin area. No further data transformation or normalization was performed. Quantitative data are presented as mean ± standard deviation. Sample sizes (n) are provided in the corresponding figure or table captions and Results sections. Each measured outcome was analyzed separately. Mechanical‐property, intradermal and transdermal drug‐delivery, and skin electrical resistance data were analyzed using Welch's one‐way ANOVA followed by Dunnett's T3 post hoc multiple‐comparisons test to compare each experimental group with the corresponding control group. Pairwise comparisons were two‐sided, and statistical significance was defined as an adjusted *p* < 0.05, corresponding to an alpha level of 0.05. Adjusted *p*‐values are reported. No inferential statistical comparisons were performed for the dimensional‐characterization data. No statistical comparison was performed for transdermal tacrolimus delivery because the values were below the detection limit in all groups.

## Author Contributions

**Aydin Sadeqi**: conceptualization, data curation, formal analysis, investigation, methodology, validation, visualization, writing – original draft. **Gulcin Arslan Azizoglu**: investigation, methodology, writing – review & editing. **Mark R. Prausnitz**: conceptualization, formal analysis, funding acquisition, methodology, project administration, supervision, writing – review & editing.

## Conflicts of Interest

M.R.P. is an inventor of patents, a paid advisor to companies, and a founder/shareholder of companies developing STAR particle technologies products, including Aldena Therapeutics, Alys Pharmaceuticals, Microstar Biotech, and Vimela Therapeutics. This potential conflicts of interest is managed by Georgia Institute of Technology.

## Supporting information



Supporting Information is available from the Wiley Online Library.
**Supporting File 1**: adhm71569‐sup‐0001‐SuppMat.pdf.

## Data Availability

The data that support the findings of this study are available from the corresponding author upon reasonable request.
