# Supplementary material for: Water‐Soluble, Enzyme‐Degradable, and Hydrolyzable STAR Particles for Enhanced Drug Delivery to Skin
Source: Adv Healthc Mater. 2026 Sep 9;15(36):e71569. doi: 10.1002/adhm.71569 (PMC13612932; doi:10.1002/adhm.71569)
Supplement: Supplementary file 1 [file ADHM-15-0-s001.pdf]

Supporting Information

Water-soluble, Enzyme-degradable, and Hydrolyzable STAR Particles for Enhanced Drug Delivery to Skin

Authors: Aydin Sadeqi, Gulcin Arslan Azizoglu, Mark R. Prausnitz*

***Corresponding Author:** Mark R. Prausnitz (*Email address:* prausnitz@gatech.edu)

Table S1. Dimensional characteristics of PVA, CA, PLA, and titania STAR particles.[1]

	**PVA STAR particles**	**CA STAR particles**	**PLA STAR particles**	**Titania STAR particles**
**Outer diameter, a (µm)**	1079 ± 14	1111 ± 29	1044 ± 23	893 ± 43
**Middle diameter, b (µm)**	226 ± 2	234 ± 4	228 ± 4	164 ± 9
**Inner diameter, c (µm)**	163 ± 16	148 ± 11	164 ± 6	140 ± 8
**Arm height, h (µm)**	497 ± 44	496 ± 7	464 ± 14	403 ± 25
**Tip diameter, tip (µm)**	22 ± 7	20 ± 3	33 ± 9	17 ± 2
**Visual appearance/color**	Translucent	Transparent	Transparent	Yellow

[1] Dimensional data are presented as mean ± standard deviation (SD), with n = 15 individual STAR particles analyzed per material. Visual appearance and color were assessed qualitatively. No inferential statistical comparisons were performed.

Table S2. Skin puncture performance of freshly made and one-week-old PVA STAR particles in different vehicles.*

	**Aloe Vera Gel**	**Mineral Oil**	**Olive Oil**	**Propylene Glycol**	**Isopropyl Palmitate**	**Isopropyl Myristate**	**Polyethylene Glycol**
**Fresh PVA STAR particles**							
**One-week-stored PVA STAR particles**							

No skin puncture — Maximum skin puncture

*Performance was evaluated based on the ability of STAR particles to puncture porcine skin *ex vivo* after rubbing on the skin for 10 s in a formulation containing 10% (w/v) STAR particles in the indicated vehicle. Week-old STAR particles were suspended in the indicated vehicle and stored for one week at room temperature before use. Skin puncture performance was qualitatively assessed based on the extent and intensity of gentian violet staining observed on porcine skin after STAR particle application. Greater staining was interpreted as increased skin puncture and assigned higher performance scores, with maximum skin puncture corresponding to that achieved by titania STAR particles applied in an aqueous vehicle (e.g., Figure 4v in the main manuscript).

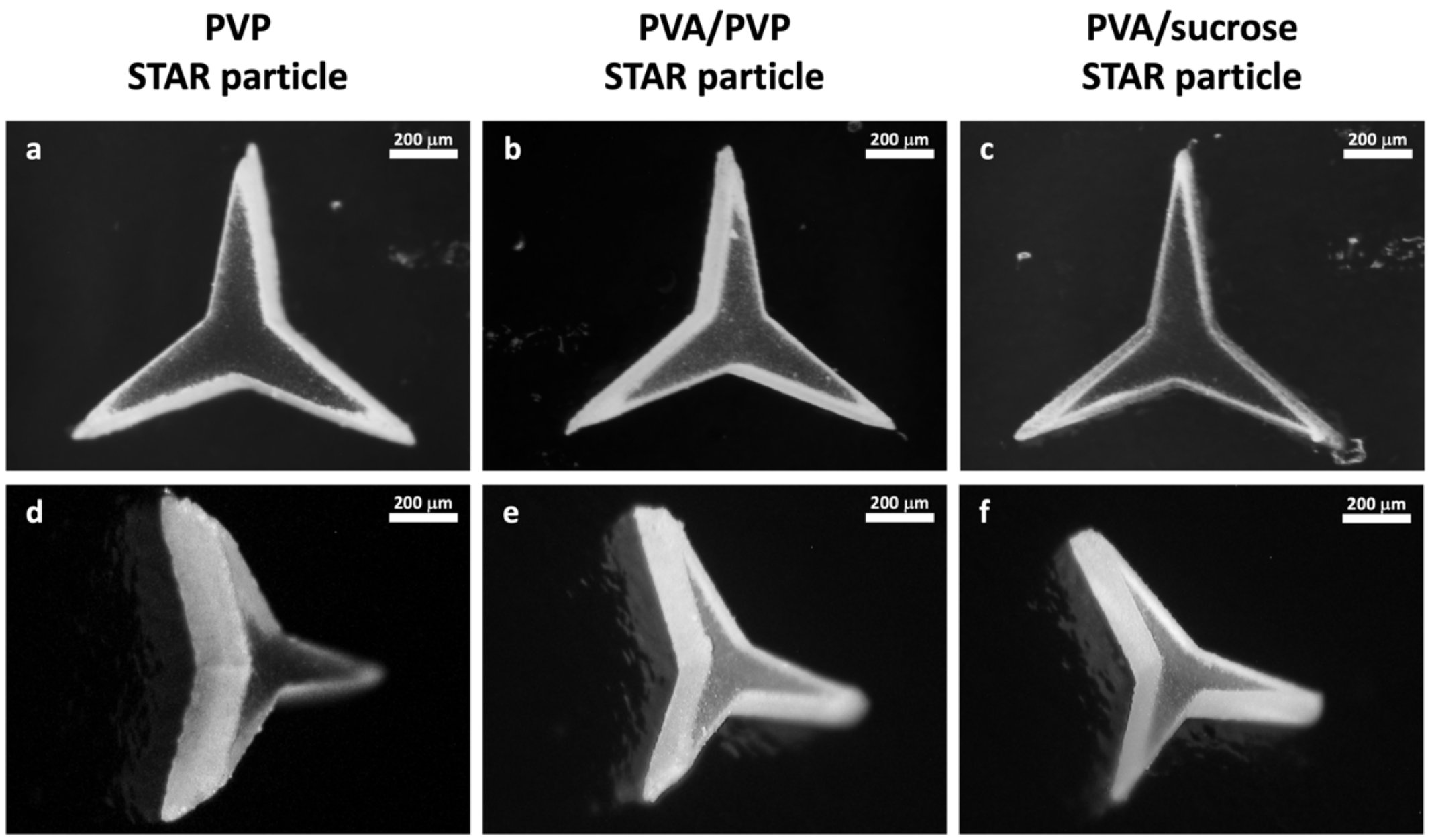

Figure S1. STAR particles made from PVP, PVA/PVP, and PVA/sucrose. Top view of representative STAR particles made of **a)** PVP, **b)** PVA/PVP, and **c)** PVA/sucrose. Side view of representative STAR particles made of **d)** PVP, **e)** PVA/PVP, **f)** PVA/sucrose. PVA/PVP and PVA/sucrose STAR particles had a formulation ratio of 50/50 (w/w).

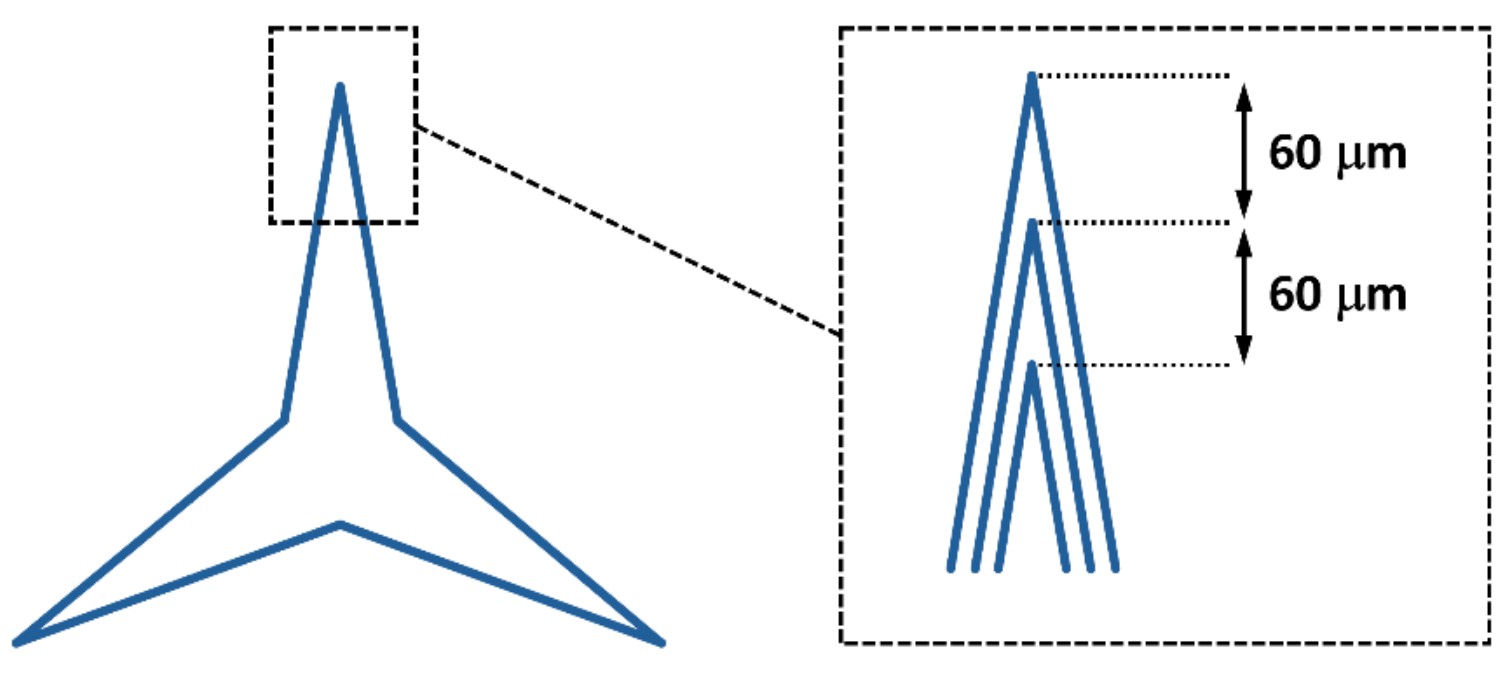

Figure S2. Two-dimensional CAD design of STAR particles used to create a tapered structure. The STAR particle design had three separate and parallel traces with a corner spacing of 60 µm to form the tapered STAR structures.
